# The effects of staged intra-articular injection of cultured autologous mesenchymal stromal cells on the repair of damaged cartilage: a pilot study in caprine model

**DOI:** 10.1186/ar4309

**Published:** 2013-09-20

**Authors:** Hui Yin Nam, Puvanan Karunanithi, Wagner Cheng Poh Loo, Sangeetha Vasudevaraj Naveen, Hui Cheng Chen, Paisal Hussin, Lucy Chan, Tunku Kamarul

**Affiliations:** 1 Tissue Engineering Group, NOCERAL, Department of Orthopaedic Surgery, Faculty of Medicine, University of Malaya, 50603 Kuala Lumpur, Malaysia; 2 Faculty of Veterinary Medicine, University Putra Malaysia, 43400 Serdang, Selangor, Malaysia; 3 Department of Orthopaedic, Faculty of Medicine and Health Sciences, University Putra Malaysia, 43400 Serdang, Selangor, Malaysia; 4 Department of Anaesthesiology, Faculty of Medicine, University of Malaya, 50603 Kuala Lumpur, Malaysia

## Abstract

**Introduction:**

Treatment of chondral injuries remains a major issue despite the many advances made in cartilage repair techniques. Although it has been postulated that the use of marrow stimulation in combination with cell-based therapy may provide superior outcome, this has yet to be demonstrated. A pilot study was thus conducted to determine if bone marrow derived mesenchymal stromal cells (BM-MSCs) have modulatory effects on the repair outcomes of bone marrow stimulation (BMS) techniques.

**Methods:**

Two full-thickness chondral 5 mm diameter defects were created in tandem on the medial condyle of left stifle joints of 18 Boer caprine (N = 18). Goats were then divided equally into three groups. Simultaneously, bone marrow aspirates were taken from the iliac crests from the goats in Group 1 and were sent for BM-MSC isolation and expansion *in vitro*. Six weeks later, BMS surgery, which involves subchondral drilling at the defect sites, was performed. After two weeks, the knees in Group 1 were given autologous intra-articular BM-MSCs (N = 6). In Group 2, although BMS was performed there were no supplementations provided. In Group 3, no intervention was administered. The caprines were sacrificed after six months. Repairs were evaluated using macroscopic assessment through the International Cartilage Repair Society (ICRS) scoring, histologic grading by O’Driscoll score, biochemical assays for glycosaminoglycans (GAGs) and gene expressions for aggrecan, collagen II and Sox9.

**Results:**

Histological and immunohistochemical analyses demonstrated hyaline-like cartilage regeneration in the transplanted sites particularly in Group 1. In contrast, tissues in Groups 2 and 3 demonstrated mainly fibrocartilage. The highest ICRS and O’Driscoll scorings was also observed in Group 1, while the lowest score was seen in Group 3. Similarly, the total GAG/total protein as well as chondrogenic gene levels were expressed in the same order, that is highest in Group 1 while the lowest in Group three. Significant differences between these 3 groups were observed (*P* <0.05).

**Conclusions:**

This study suggests that supplementing intra-articular injections of BM-MSCs following BMS knee surgery provides superior cartilage repair outcomes.

## Introduction

Chondral injuries often afflict the young owing to their athletic lifestyle and the high impact mechanical loading subjected to their joints during routine activities [1]. Injuries, especially those which result in focal cartilage defects, lead to an immediate loss in articular surface smoothness, resulting in the increase in tissue attrition. This triggers a series of changes to the subchondral bone which can lead to joint pain and dysfunction which, if left untreated, can lead to osteoarthritis [2]. Although in rare cases, it has been reported that the full-thickness chondral defect can heal spontaneously, the resultant repair tissue forms fibrous cartilage which will eventually lead to tissue degeneration [3]. This poses serious issues, as many of these patients would present with irreversible cartilage damage by the time they seek help from health care providers. The urgency to resolve this problem becomes more apparent when such conditions involve the young with many years of productivity still expected of them [4,5].

To halt the progress of cartilage deterioration as the result of focal cartilage damage, several conventional treatments have been suggested, including marrow stimulation procedures [6]. This technique involves creating channels through the subchondral bone that allow access for blood and marrow elements to reach the damaged surface, which have been suggested to contain the essential ingredients by which cartilage healing occurs [7]. However, the superclot that forms in the defect as it matures with time tends to develop into a mixture of fibrocartilage and hyaline-like repair tissue. Nevertheless, using this technique, Steadman *et al*. were still able to demonstrate that bone marrow stimulation (BMS) can produce good outcomes in short- to mid-term follow-up of patients who had undergone this procedure [8]. This success, however, is short-lived and, thus, further improvements to this technique may lead to better long-term outcomes.

In recent years, it has been suggested that the use of intra-articular injection of mesenchymal stromal cells (MSCs) may result in moderately good repair outcomes, even in joints that have undergone mild degeneration [9]. In several studies, it has been shown that MSCs in circulating synovial fluid not only restores cartilage integrity but also halts the progression of cartilage degeneration [10]. In both BMS and MSC injection to the joint, there have been many suggestions to the mechanisms involved that have resulted in the positive outcomes observed; however, none have been sufficient to provide a satisfactory or conclusive explanation [11]. It may be the case that the combination of both techniques may result in a synergistic outcome, thus providing superior tissue repair. Based on our extensive literature search, there have not been any published results that support this notion, although one study using mononuclear cells and not MSCs suggests that this may be the case [12]. To prove whether such a synergistic effect exists, a study was conducted using a focal cartilage defect in a caprine model treated with BMS with or without intra-articular injection of MSCs. Outcome measures were conducted six months post-operative using macroscopic, histology, and selective cartilage protein and gene expression assessments.

## Methods

### Animals

Ethical approval for animal research was obtained from the Institutional Animal Care and Committee (IACUC) in the Faculty of Veterinary Medicine, University Putra Malaysia (Ref no: UPM/FPV/PS/3.2.1.551/AUP-R89), and the Faculty of Medicine, University of Malaya (Ref no: OS/14/10/2009/NHY (R)). The outcome parameters of 18 adult caprines of the Boer breed (*Capra aegragus hircus*) were compared in this study. The average age and weight of the goats used were 1.5 years (1.2 to 1.8 years) and 33.7 kg (28.8 kg to 38.8 kg), respectively, at the beginning of the study. Animals were held in big indoor runs for at least one week in order to acclimatize them to the surroundings, food was unrestricted and water was available *ad libitum* prior to starting the experiment. Animals were randomized into three groups based on the type of intervention performed. The groups and interventions performed are summarized in Table 1. Only the animals from Group 1 underwent bone marrow aspiration from the left iliac crest region in order to obtain the BM-MSC to be used two weeks after BMS.

**Table 1:** Study design

**Group**	**Number**	**Treatment**
1	6	Supplement with autologous BM-MSCs
2	6	Single negative (BMS, no supplement)
3 (control)	6	Double negative (no BMS, no supplement)

### Harvesting and isolation of caprine MSCs

General anesthesia by intravenous ketamine and diazepam injection were used in this study. Bone marrow was aspirated from the goats using biopsy needles. Bone marrow aspirates were then placed in syringes containing heparin (5,000 U) and kept on ice throughout the transportation to the laboratory. Aspirated bone marrow was added to an equal volume of pH 7.2 phosphate-buffered saline (PBS) (Invitrogen-Gibco, Grand Island, NY, USA) and layered on the density of 1.077 g/mL Ficoll-Paque (Amersham Biosciences, Uppsala, Sweden). Centrifugation at 2,200 rpm for 25 minutes was then performed. The mononuclear cells were isolated and resuspended with 10 ml low-glucose Dulbeccoo’s modified eagle medium (L-DMEM) through centrifugation at 1,600 rpm for 10 minutes. The supernatant was then discarded, and the cell pellets were cultured in growth medium (low-glucose DMEM supplemented with 10% fetal bovine serum, 100 U/mL penicillin, 100 μg/mL streptomycin and 1% GlutaMAX-1; Invitrogen-Gibco, USA) in T-75 tissue culture flasks. At three to four days after culture, fresh growth medium was replaced every three days until the cultures became 75% to 80% confluent. The cells were serially passaged and expanded up to passage-2 before being used for transplantation.

### *In vitro* lineage differentiation

The multipotent capacity of caprine MSCs was proven after *in vitro* culturing with specific supplements by inducing differentiation into osteogenic, chondrogenic and adipogenic phenotypes with triplicate cultures, respectively. To induce osteogenic differentiation, confluent passaged-3 cells were cultured in the osteogenic medium (Invitrogen-Gibco, USA). After 21 days, Alizarin Red staining was used to observe the matrix mineralization. For adipogenesis, adipogenic medium (Invitrogen-Gibco, USA) was used to induce the differentiation in the confluent culture of passaged-3 cells. Fourteen days after culture initiation, the cells were fixed with methanol at room temperature for 10 minutes, rinsed by 60% isopropanol, and stained by using freshly prepared Oil Red O solution in 99% isopropanol for 15 minutes. Chondrogenic differentiation was induced using a micromass culture system. For this purpose, 1 × 10^6^ passaged-3 cells were pelleted under 1,800 rpm for five minutes and cultured in a chondrogenic medium (Invitrogen-Gibco, USA). Twenty-eight days after initiation of the culture, the pellets were removed and subjected to the following: fixing in 10% formalin for one hour; dehydrating in ascending concentrations of ethanol; clearing in xylene; embedding in paraffin wax and sectioning at 4 μm using a microtome. The sections were then stained, using Safranin O for five minutes at room temperature.

### Expression of surface markers

To ensure that the isolated cells consist of the homogeneous population of the defined MSCpopulation, expression of surface markers in caprine MSC cultures at passage-3 was performed using immunohistochemistry staining. Cells were seeded at 10,000 cells per chamber in the four-well chamber slides and the protocol was performed according to the manufacturer’s recommendation (Dako, Glostrup, Denmark). Briefly, MSCs were fixed in 4% paraformaldehyde/PBS for 15 minutes, and then blocked for 30 minutes using hydrogen peroxidase (H_2_O_2_) to prevent endogenous activity. MSCs were then incubated in goat serum working solution for 15 minutes to block non-specific binding. MSCs were incubated with primary antibody (rabbit anti-goat CD44^+^/CD29^+^ and CD45^-^/CD34^-^ monoclonal antibody, 1:100 dilution, Abcam, Kendall, Sq, Cambridge, UK) at room temperature for 30 minutes. After washing with PBS, cells were incubated with secondary antibody (goat anti-rabbit IgG, Dako) at 1:200 dilution for 30 minutes. Cells were then washed with PBS, stained with 3,3′-diaminobenzidine tetrahydrochloride (DAB) chromogen substrate and examined under light microscopy (Nikon Eclipse TE2000-S; Nikon Corporation, Tokyo, Japan).

### Defect localization and surgery

**Figure 1:**
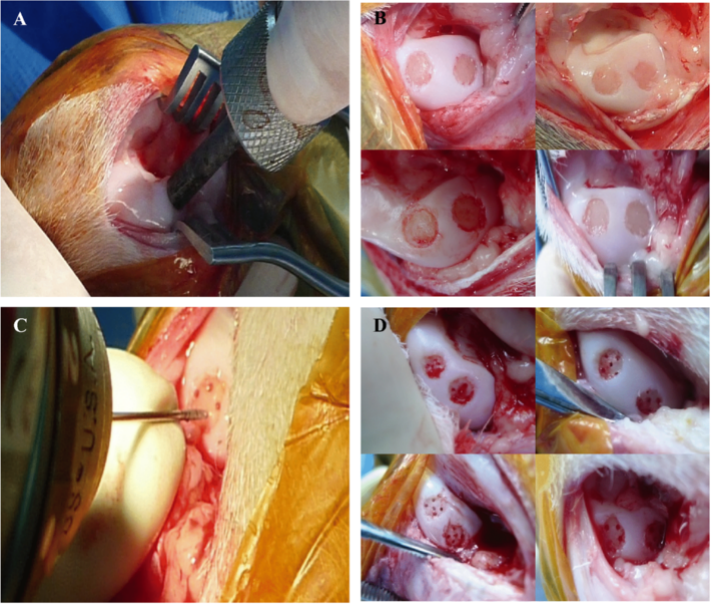
**Full thickness chondral defects. (A)** Chondral defects with 5 mm diameter chondrotome. **(B)** Removal of calcified cartilage layer with curette, leaving an intact subchondral bone plate. **(C**,**D)** Five drill holes per chondral defect can be clearly observed after being washed with saline.

Surgeries were performed under aseptic conditions. Following the administration of general anesthesia, the knee joints were surgically approached through medial parapatellar incisions. The articular surfaces were exposed by laterally dislocating the patellae. Full-thickness chondral defects measuring 5 mm diameter (Figure 1B) were created on the medial condyle of the left knee joint of the caprines using a custom-made cylindrical chondrotome (Figure 1A). A similar defect was also created at a site slightly posterior to the one previously created. Any bone or cartilage debris was removed carefully either physically or using copious amounts of normal saline. The wound was closed in layers.

Six weeks after the surgery, the defects were subjected to BMS using 0.9 mm diameter Kirschner wires, to a depth of approximately 6 mm to 8 mm (Figure 1C), or when bleeding was observed from the drill holes. This technique was similar to that described by Steadman *et al*. [7]. The holes were created at the periphery of the defects, close to the defect walls (to enhance repair tissue integration) and then advanced to the center, keeping the holes well-spaced at 1 mm to 2 mm to prevent propagation of holes and collapse of the subchondral bone (Figure 1D).

### Intra-articular injection of caprine MSCs

Two weeks after BMS, the caprines in Group 1, received a weekly intra-articular injection of autologous MSCs (1 × 10^7^ cells) for three consecutive weeks. Injections were performed using large 18G size needles to avoid lysis of cells.

### Macroscopic evaluation

Animals were euthanized at 29 weeks after the initial stage of cartilage defect creation. The knee joints were dissected to obtain the distal femur. Gross inspection of the repaired chondral defects were performed simultaneously. Two independent examiners who were blinded to the different groups were asked to examine the knee joints. The joints were photographed, recorded and assessed following the modified component of the International Cartilage Repair Society (ICRS) Cartilage Repair Assessment scoring scale (macroscopic appearance subcategory) [13]. Upon completion of the scoring, the specimens were halved using a mechanical bone saw (Fein MultiMasterAccu, C&E Fein Gmbh, Stuttgart, Germany). The specimens were then sampled accordingly, in order to perform several analyses which meant that samples were: a) fixed in 10% phosphate-buffered formalin for histology and immunostaining, b) tested for glycosaminoglycans (GAGs) content and, c) tested using real-time polymerase chain reaction (RT-PCR) to determine selected gene expression levels.

### Histologic examination and immunohistochemical staining

The specimens were fixed in 10% buffered formalin overnight and then decalcified using 10% formic acid. The specimens were subsequently dehydrated in ethanol in a stepwise manner from 70% up to 100%, transferred to xylene and embedded in paraffin. A total of 4 μm paraffin sections were prepared longitudinally. The samples were stained with hematoxylin and eosin to evaluate the cellular architecture, and Safranin O-Fast Green was used to detect cartilage proteoglycan. Immunohistochemical staining was performed using DAKO EnVision 1 System peroxidase kit (DAKO, Denmark). To detect the collagen distribution in the repaired tissue, the specimen section slides were incubated in primary antibody solution (Calbiochem-Daiichi Fine Chemical Co., Takaoka, Toyama, Japan) followed by secondary antibody and substrate-chromogen solution following the instructions provided by the manufacturer. Histological specimens were scored following the scale provided by the modified O’Driscoll histological cartilage scoring system [14] (Table 2). Histological analysis of the repaired tissue was performed for each specimen by observers who were blinded to the sample origin. Special attention was made to the overall appearance of the repaired tissue, cell shape, the extent of defect filling, and the integration of the newly formed tissue with the defect edges.

**Table 2:** Modified O’Driscoll histological and histochemical grading scale

	**Score**
**Nature of the predominant tissue**	
**Cellular morphology**	
**Hyaline articular cartilage**	4
**Incompletely differentiated mesenchyme**	2
**Fibrous tissue or bone**	0
**Safranin-O staining of the matrix**	
**Normal or nearly normal**	3
**Moderate**	2
**Slight**	1
**None**	0
**Structural characteristics**	
**Surface regularity**	
**Smooth and intact**	3
**Superficial horizontal lamination**	2
**Fissures - 25 to 100% of the thickness**	1
**Severe disruption, including fibrillation**	0
**Structural integrity**	
**Normal**	2
**Slight disruption, including cysts**	1
**Severe disintegration**	0
**Thickness**	
**100% of normal adjacent cartilage**	2
**50 to 100% of normal cartilage**	1
**0 to 50% of normal cartilage**	0
**Bonding to the adjacent cartilage**	
**Bonded at both ends of graft**	2
**Bonded at one end, or partially at both ends**	1
**Not bonded**	0
**Freedom from cellular changes of degeneration**	
**Hypocellularity**	
**Normal cellularity**	3
**Slightly hypocellularity**	2
**Moderate hypocellularity**	1
**Severe hypocellularity**	0
**Chondrocyte clustering**	
**No clusters**	2
**<25% of the cells**	1
**25 to 100% of the cells**	0
**Freedom from degenerative changes in adjacent cartilage**	
**Normal cellularity, no clusters, normal staining**	3
**Normal cellularity, mild clusters, moderate staining**	2
**Mild or moderate hypocellularity, slight staining**	1
**Severe hypocellularity, poor or no staining**	0

### Quantitative analysis of cartilage repair glycosaminoglycans (GAGs)

Protein and glycosaminoglycan (GAGs) were determined using a Bio-Rad DC protein assay kit (Bio-Rad Laboratories, Alfred Nobel Drive, Hercules, CA) and Blyscan sulfated glycosaminoglycan assay kit (Biocolor Ltd, County Antrim). The lesions within different groups were pooled for this analysis. Specimens were dissected and digested using RIPA buffer (Merck & Co, Whitehouse Station, NJ, USA) supplemented with protease inhibitors (Sigma-Aldrich, St Louis, MO) for 30 minutes, following the manufacturer’s protocol. Spectrophotometer absorbance measurements were performed at 750 nm and 656 nm for protein and GAGs assays, respectively. GAGs content was normalized with the total protein contents (μg GAGs/g protein).

### Total RNA extraction, cDNA synthesis and real-time PCR

Total RNA was isolated using a homogenizer and then processed according to the cartilage RNA isolation kit (Biochain Institute, Breakwater Avenue, Hayward, USA) protocol. RNA samples were finally re-dissolved in 30 μl water and stored at -20°C. One μg of RNA was used to generate cDNA with the Superscript III first strand synthesis kit (Invitogen-Life Technologies, Carlsbad, CA, USA) following the manufacturers’ instructions. Real-time PCR analysis (CFX96 Real-time system, Bio-Rad) was performed to assess the mRNA levels using iQ-SYBR green supermix (Bio-Rad). The data were normalized using glyceraldehyde-3-phosphate dehydrogenase (GAPDH). The primers used for real-time PCR are summarized in Table 3.

**Table 3:** RT-PCR primers sequences (1st BASE Pte. Ltd., Singapore)

**Gene**	**RT-PCR primer sequences**	**Tm (°C)**	**GC (%)**
Collagen II (Forward)	5′-CTG GAT GCC ATG AAG GTT TT-3′	58.4	45.0
Collagen II (Reverse)	5′-TCT TGT CCT TGC TCT TGC TG-3′	60.4	50.0
Aggrecan (Forward)	5′-GCA AGT GGT CTT CCT TCT GG-3′	62.4	55.0
Aggrecan (Reverse)	5′-TTC CAC CAA TGT CGT ATC CA-3′	58.4	45.0
Sox 9 (Forward)	5′-TGA AGA AGG AGA GCG AGG AG-3′	62.4	55.0
Sox 9 (Reverse)	5′-GAC GTG CGG CTT GTT CTT-3′	59.9	55.6
GAPDH (Forward)	5′-GCT CTC TTC CAG CCT TCC TT-3′	62.4	55.0
GAPDH (Reverse)	5′-TAG AGG TCC TTG CGG ATG TC-3′	62.4	55.0

GC (%), GC content; Tm (°C), Melting temperature.

### Statistical analysis

The overall differences for each parameter were determined using non-parametric analyses, that is, Kruskal-Wallis and Mann Whitney U tests, to evaluate the level of significance between the groups. *P-*values of less than 0.05 were considered significant. Statistical analyses were performed using SPSS version 17 (SPSS Inc, Chicago, IL, USA).

## Results

### Cell culture

**Figure 2:**
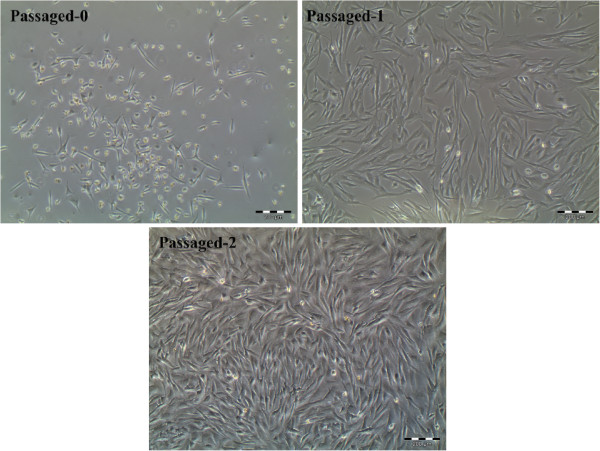
**Caprine bone marrow cell culture.** At primary culture passage-0 (Day 6), fibroblastic as well as small clear cells can be observed. The number of clear cells was decreased during the passages and fibroblastic cells became dominant in culture.

The primary cultures of the goat bone marrow cells contained mainly fibroblastic cells as well as a few small round cells. Rounded cells appeared to be reduced in numbers in subsequent subcultures. It is likely that only fibroblastic-appearing MSCs were left at the end of cell cultures (Figure 2).

### Characterization of caprine MSCs

#### Immunophenotyping analysis

**Figure 3:**
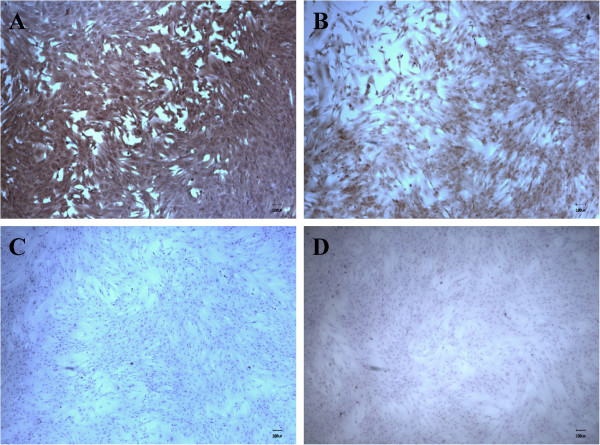
**Immunophenotyping of caprine MSCs for expression of multiple CD antigens. (A)** CD 29, **(B)** CD 44, **(C)** CD 45, and **(D)** CD 34.

The cells showed an abundance of CD29 and moderate CD44 expressions (Figure 3), which are generally accepted markers for MSCs [15]. In contrast, no expression of the hematopoietic lineage markers CD45 and CD 34 was observed in the cultures.

#### Multi-lineage differentiation

**Figure 4:**
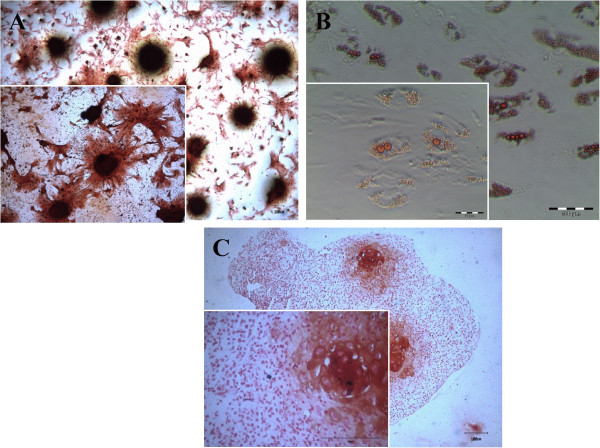
**Tri-lineage differentiation potential of primary MSCs. (A)** Osteogenic differentiation. **(B)** Adipogenic differentiation. **(C)** Chondrogenic differentiation.

In osteoinductive cultures, nodule-like structures were observed in certain regions. Using alizarin red staining, red-mineralizing areas were apparent, indicating that the cells were in the early stages of bone formation (Figure 4A). In adipogenic culture, small lipid droplets appeared within the cytoplasm of the cells on days 3 and 4. They gradually occupied whole cells by Day 14. The lipid droplets turned red when stained by the Oil Red O staining (Figure 4B). In the micro-mass culture system for chondrocyte differentiation, the size of the pellet seemed to be increased during the culture period, probably as a result of matrix production and secretion. The metachromatic nature of the matrix was demonstrated by the Safranin O staining (Figure 4C). These observations suggested that MSCs have the potential to undergo tri-lineage differentiations, including chondrogenic, osteogenic and adipogenic differentiation [16].

### Macroscopic observations

No signs of osteoarthrosis, such as osteophytes, cyst formation, cartilage erosion or synovial proliferation, were observed in any of the knees. At 29 weeks, the typical macroscopic appearance of the MSC-treated specimens, that is, Group 1, indicated marked improvement of the filling of the defects as compared with the other groups (figures not provided). In this group, healing was almost complete and the reparative tissue produced appeared similar to normal cartilage. There also appeared to be good integration of tissue at the margins of the repair site, with flush and smooth surfaces, and good thickness observed on the repaired cartilage. Partial filling of the defect was seen in the BMS group, that is, Group 2, with clear apparent edges and irregular surfaces. No healing was seen in the control group and, the margins of the defects were clearly distinguishable with minimal filling of the reparative tissue.

**Figure 5:**
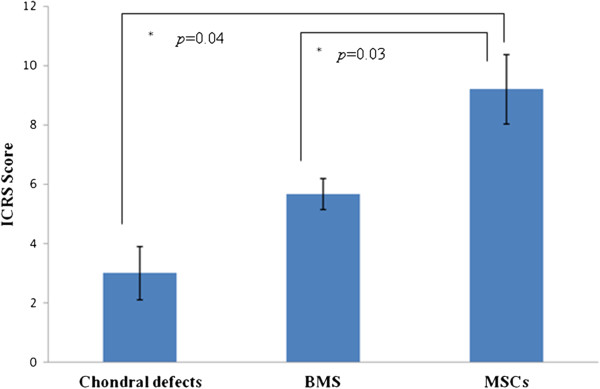
**Macroscopic ICRS scoring of chondral defect repair.** (Significance is represented by *).

These macroscopic observations were scored using the ICRS visual scoring system (Figure 5). Overall, the MSC-treated group had the most consistently good cartilage repair, whereas the untreated group had the worst overall repair. Significant differences were observed between the MSC-treated group (mean score = 9.20 ± 1.16) and the BMS group (mean score = 5.67 ± 0.52; *P* <0.05) for ICRS score. These scores were significantly different than the control group which has a mean score of 3.00 ± 0.89 (*P* <0.05).

### Histological observations

**Figure 6:**
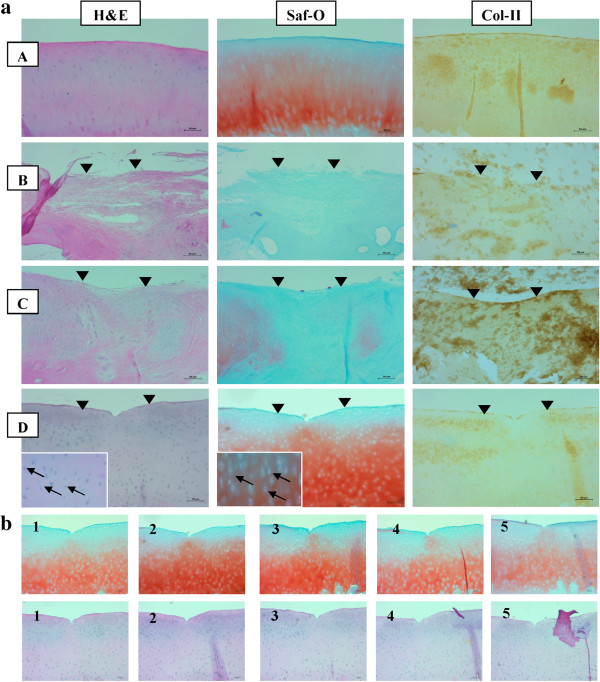
**Histological images at the 29**^**th **^**week. a. (A)** Normal knee, **(B)** Chondral defect, **(C)** Defects treated with BMS, **(D)** Defect treated with autologous MSCs. (defect region s represented by ▼). **b.** Different images of histological staining (Saf-O and H&E) from the edge to the central lesion (1 to 5).

The microscopic appearance of the tissue using various staining methods revealed cartilage regeneration within the treated sites. There was marked improvement in the quality of the repaired tissue in the MSC-treated group when compared to the other groups (Figure 6). The tissue was hyaline-like, with good integration, thickness and surface regularity. In contrast, the untreated defects did not appear to undergo complete healing. In the hematoxylin and eosin-stained sections, the MSC-treated group showed substantial thickening of the cartilage tissue when compared with the other groups. Most of the chondrocytes of this group had an appearance comparable with that of hyaline cartilage. At higher magnification, the cells resembled well-differentiated chondrocytes which were surrounded by metachromatic matrix (Figure 6). The regenerated tissues from MSC-treated groups showed a continuous surface with a mixture of hyaline and fibrocartilage. Furthermore, the MSC-treated tissue sections showed relatively heavier staining with Safranin O, suggesting a higher concentration of proteoglycans, which is one of the major components of cartilage tissue. Immunohistochemical staining for type II collagen further confirmed that MSC-treated tissue sections showed a relatively higher expression of cartilaginous collagen throughout the whole neocartilage as compared with other treated groups. This distribution pattern is similar to that of the normal cartilage.

**Figure 7:**
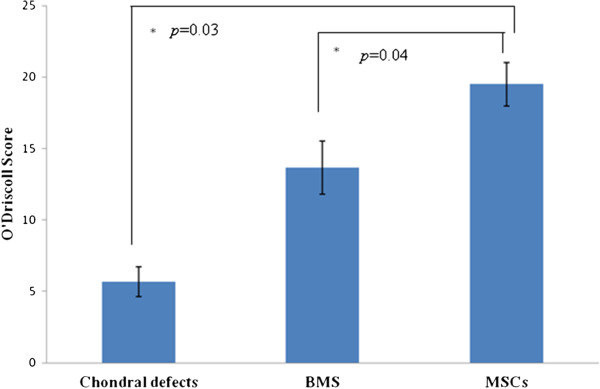
**Quantitative histologic evaluation of the regenerated cartilage using O’Driscoll scores.** (Significance is represented by *).

O’Driscoll histological scores (Figure 7) of the post-implantation repair tissue was significantly (*P* <0.05) higher in the transplanted sites of the MSC treated group (19.50 ± 1.52), than that for the group BMS (13.67 ± 1.86) and the control group (5.67 ± 1.0333).

### Biochemical analysis

**Figure 8:**
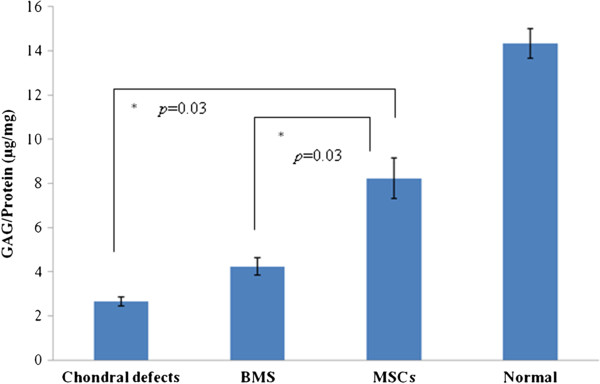
Comparison in the glycosaminoglycan/protein content in the pooled lesions within the different groups.

Consistent with the morphological and histological scores, the mean levels of GAG/protein (μg/mg) were higher in the MSC-treated knees (8.24 ± 0.94) than the BMS (4.25 ± 0.42) and the defect groups (2.65 ± 0.22) (Figure 8). A significant difference in GAGs/protein content was observed between all groups when compared with normal caprine cartilage.

### Gene expression analysis

**Figure 9:**
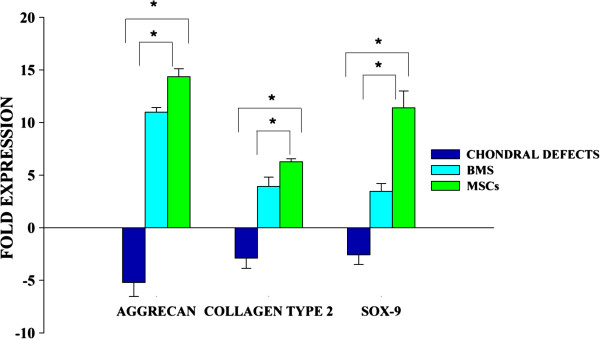
Gene expression analyses of aggrecan, collagen II and SOX9 demonstrates variations between groups.

Comparative analysis of cartilage gene expressions for all the groups is shown in Figure 9. Significant differences were observed among all groups in aggrecan and SOX-9 (*P* <0.05). Although significant differences were observed in the expression of type II collagen between the Groups 1 or 2 vs. Group 3, no significant difference was observed between the treated groups, that is, Groups 1 and 2.

## Discussion

In the present study, the potential of autologous BM-MSCs was compared to a standard treatment for focal cartilage defect, that is, subchondral drilling as the method for marrow stimulation, in caprine models. Data from the present study suggest that the use of BM-MSCs as an adjunct therapy provides certain levels of improvements to this method of cartilage repair.

Although the authors of the present paper are only aware that the present study conducted is the first to demonstrate such findings, there were several previous studies using almost similar techniques that are worth comparing. In 2009, Saw *et al.*[12] described the use of bone marrow aspirates and hyaluronic acid (HA) in improving the repair of BMS-treated chondral defects. In their experimental study, they found that from using this technique the cartilage repair produced integrated hyaline-like tissue. This led to their claim that there may be a redundancy of practicing MSCs’ isolation and *in vitro* expansion, as marrow aspirates may be sufficient to achieve good results. However, it is noteworthy that the limitations in this study were obvious. Among these was the fact that the conclusion was based on a single histological score using four goats in each group. It was statistically weak and lacked sufficient measured parameters. The authors then published a cohort study involving 50 patients but this time using peripheral derived stem cells [17]. They claim that the outcome was good, based on limited representative histological scoring and MRI only. However, there are several issues that this paper did not address, and this could have led to the over-rated findings of that reported study. While the authors mentioned that the cells obtained from the peripheral blood were stem cells, there was no evidence to support such claims since there was no cellular characterization performed. Furthermore, the cells obtained do not fulfill any criteria that identify them as stem cells, that is, the ability for higher cell potency, such as multipotency. With no cell sorting or selection performed, at best the cells used can be described as mixed mononuclear cells. There also appears to be a discrepancy between the histological and patient outcome scores which does not appear to correlate, demonstrating that the outcome may not have been as good as what has been concluded. The issue in this study, and that of others, would have been whether histological scores reflect the good outcomes that are measured following any tissue repair. In many of the studies we reviewed, although histological analyses were used, other parameters were also measured which include patient scores, protein and/or gene expression, and functional biomechanical analyses [18,19]. In these studies, correlations between histological scores and the final outcomes were always present. This is similar to what was found in our study. There was strong correlation found between these scores and that each of the measured parameter appears to corroborate the findings of the other parameters. We can, therefore, safely assume that the reports from our study are more likely to reflect better accuracy and thus are more reliable as compared to the study reporting the use of marrow or peripheral blood mononuclear cells.

In a separate study by Lee *et al*., chondral defects treated using bone marrow MSCs only, contributed to superior cartilage repair [20]. This seems to support our findings that when a combination of MSCs and BMS is used, a superior outcome can be expected. Unlike the previous study by Saw *et al.,* which lacked characterization to claim that the cells were stem cells, the study by Lee *et al*. demonstrated several features which suggest that the cells used were as such, albeit limited in terms of today’s standard since no surface CD marker expressions were investigated in their study. One advantage their study offered over the presently reported article is that observations were carried out in two different time points, which showed progressive improvements. In contrast, ours, which lasted up to 29 weeks, merely showed good repair outcomes in the treated group. One may argue that given a larger time scale, those goats not supplemented may have had comparable outcomes. Nevertheless, if no improvements were observed at six months, we can safely say that a treatment has most likely reached its ultimate endpoint of progression.

There were several studies that used intra-articular injection of stem cells; however, these involved smaller animals without marrow stimulations and are usually osteoarthritic models as opposed to focal cartilage defects. It is worth noting that osteoarthritis represents a degenerative stage which is usually the end stage of a diseased condition, while focal cartilage defects represent the pre-degenerative state at best. Among the studies worth mentioning is that by Agung *et al*. [21], which evaluated the use of MSCs in rodents. It was demonstrated that using MSCs injected into injured knee joints could mobilize these cells to the injured area, contributing to tissue regeneration. Similarly, this was observed by our co-researchers [10]. They examined the effects of MSCs, HA and the combination of HA-MSCs in treating osteoarthritis (OA) in a rat model. Their study suggested that the use of either HA or MSCs effectively reduces OA progression better than their combined use, which suggests that MSCs have modulatory effects on damaged articular cartilage. These studies appear to suggest that the findings of our study are not unexpected as MSCs produce positive healing effects when injected intra-articularly.

The reason for the fibrous tissue formation instead of hyaline cartilage seen in the BMS only group may be explained by the inflammatory response invoked due to trauma. It is suggested that the combination of BMS and BM-MSC may have slowed this process, thereby promoting hyaline cartilage regeneration as opposed to fibrocartilage formation. The reason for this, and even for those observed in our study, is rather contentious, since there are already many scientists debating the issue as to whether the improvement seen in such cases is the direct result of the repair by the MSCs, or whether it is the factors produced by MSCs, or even perhaps the proteins they attract which ultimately result in direct repair or retardation of the inflammatory process [11]. In previous reports, MSCs are said to be able to secrete a broad spectrum of bioactive molecules that have immunoregulatory [22-24] and/or regenerative activities [25]. Bioactive factors secreted by MSCs have been shown to inhibit tissue scarring; suppress apoptosis, inflammatory and fibrotic; stimulate angiogenesis where MSCs secrete a growth factor, VEGF (Vascular Endothelium Growth Factor), which stimulates vascularization; and enhance mitosis of tissue-intrinsic stem or progenitor cells. Other studies have shown that MSCs attach to the defect sites while others have shown that in the transplanted site MSCs regress dramatically to a point that they are no longer viable [26,27]. Regardless of the mechanisms involved, the present study clearly demonstrated a correlation between the use of MSCs with superior tissue repair, which will require further investigations if we wish to elicit the mechanisms involved. It must be stressed that the data presented here were not only based on macroscopic and histological assessments, but also include other investigations, such as GAG content and selective cartilage gene expression, that is, SOX9 and Collagen II*,* which demonstrated a significant repair process as compared to BMS alone. This demonstrates the complex relationship in the healing processes involved, which justifies our claims that the use of MSCs as an intra-articular adjuvant to BMS improves the many facets of articular joint healing and that the observation made is not merely by chance.

Despite the robust findings presented here, our study presents several limitations that are worth noting. Financial restriction prohibited functional assessment of the repaired site, such as biomechanical testing, to be conducted. Thus, it was difficult to ensure whether our observation of the histologic appearance of the regenerated cartilage is directly proportional to the biomechanical function of the tissues. Nevertheless, we consider that this study still provides valuable findings since the conclusions were drawn using well-accepted experimental techniques utilized in several other previous studies [28,29]. Introduction of another treatment arm that utilizes a standard treatment approach for cartilage repair, such as hyaluronic acid [30] injection, although not mandatory, could have led to a more comprehensive conclusion. Improvement could also be made to the study by using a larger number of animals, thereby increasing the significance levels in this study. Nevertheless, one could argue that statistically this number of samples was deemed appropriate as is. It is well known that repair of articular cartilage lesions remodels with time [6,31-33]. Evaluation of the result at only one time point, at six months, is hence unable to detect progressive remodeling that has been shown to occur beyond a year [6]. This should be addressed in future studies.

## Conclusions

In conclusion, our preliminary study demonstrates that within reasonable limits, the use of supplementary intra-articular injection of BM-MSCs produced modulatory effects on the repair produced by marrow stimulation. This study, however, will require further evaluations and more convincing results using more rigorous experiments. These questions will be answered in our ongoing study, which is presently being conducted based on the results of the currently reported pilot project.

## Abbreviations

BM-MSCs: Bone marrow derived mesenchymal stromal cells; BMS: Bone marrow stimulation; CD: Cluster of differentiation; cDNA: Complementary deoxyribonucleic acid; Col-II: Collagen type II; DAB: 3,3′-Diaminobenzidine; g: gram; GAGs: Glycosaminoglycans; GAPDH: Glyceraldehyde-3-phosphate dehydrogenase; H&E: Hematoxylin and eosin; H2O2: Hydrogen peroxide; HA: Hyaluronic acid; IACUC: Institutional animal care and committee; ICRS: International cartilage repair society; kg: Kilogram; L-DMEM: Low-glucose Dulbecco’s modified eagle medium; mg: Milligram; min: Minute; ml: Milliliter; mm: Millimetre; MSCs: Mesenchymal stromal cells; nm: Nanometre; OA: Osteoarthritis; PBS: Phosphate-buffered saline; RIPA: Radio-immunoprecipitation assay; RNA: Ribonucleic acid; rpm: Revolution per minute; RT-PCR: Real-time polymerase chain reaction; Saf-O: Safranin O; Tm: Melting temperature; μg: Microgram; VEGF: Vascular endothelium growth factor.

## Competing interests

The authors declare that they have no competing interests.

## Authors’ contributions

TK, NHY, PK, LC and WLCP conceived and designed the study. NHY and PK performed cell cultures, histological, biochemical and immunohistochemical analyses. NHY, PK and SVN carried out the gene expression analyses. TK and LC performed scoring systems and image analyses. WLCP and PH performed the surgery and the administration of cells into the caprine knee joint, while CHC performed the anesthesia procedures. TK, NHY, PK and SVN helped to draft the manuscript. All authors read and approved the final manuscript.
